# Co-infection of HIV in patients with Buruli ulcer disease in Central Ghana

**DOI:** 10.1186/s12879-021-06009-7

**Published:** 2021-04-08

**Authors:** Yaw Ampem Amoako, Aloysius Dzigbordi Loglo, Michael Frimpong, Bernadette Agbavor, Mohammed Kabiru Abass, George Amofa, Elizabeth Ofori, Edwin Ampadu, Kingsley Asiedu, Ymkje Stienstra, Mark Wansbrough-Jones, Tjip van der Werf, Richard Odame Phillips

**Affiliations:** 1 School of Medicine and Dentistry, Kwame Nkrumah University of Science and Technology, Kumasi, Ghana; 2 Department of Medicine, Komfo Anokye Teaching Hospital, Kumasi, Ghana; 3 Skin NTD’s Research Group, Kumasi Centre for Collaborative Research in Tropical Medicine, Kumasi, Ghana; 4 Agogo Presbyterian Hospital, Agogo, Ghana; 5 Dunkwa Government Hospital, Dunkwa, Ghana; 6 Tepa Government Hospital, Tepa, Ghana; 7 National Buruli ulcer Control Programme, Ghana Health Service, Accra, Ghana; 8 Department of Neglected Tropical Diseases, WHO, Geneva, Switzerland; 9 Department of Medicine/ Infectious Diseases, University of Groningen, University Medical Centre Groningen, Groningen, Netherlands; 10 Institute of Infection and Immunity, St George’s University of London, London, UK

**Keywords:** Buruli ulcer, Human immunodeficiency syndrome, Coinfection, Mycolactone, Interferon-gamma, Antiretroviral therapy, Immune reconstitution syndrome, Paradoxical reaction

## Abstract

**Background:**

Previous studies have reported that presence and severity of Buruli ulcer (BU) may reflect the underlying immunosuppression in HIV infected individuals by causing increased incidence of multiple, larger and ulcerated lesions. We report cases of BU-HIV coinfection and the accompanying programmatic challenges encountered in central Ghana.

**Methods:**

Patients with PCR confirmed BU in central Ghana who were HIV positive were identified and their BU01 forms were retrieved and reviewed in further detail. A combined 16S rRNA reverse transcriptase / IS2404 qPCR assay was used to assess the *Mycobacterium ulcerans* load. The characteristics of coinfected patients (BU^+^HIV^+^) were compared with a group of matched controls.

**Results:**

The prevalence of HIV in this BU cohort was 2.4% (compared to national HIV prevalence of 1.7%). Eight of 9 BU^+^HIV^+^ patients had a single lesion and ulcers were the most common lesion type. The lesions presented were predominantly category II (5/9) followed by category I lesions. The median (IQR) time to healing was 14 (8–28) weeks in the BU^+^HIV^+^ compared to 28 (12–33) weeks in the control BU^+^HIV^−^ group (*p* = 0.360). Only one BU^+^HIV^+^ developed a paradoxical reaction at week 16 but the lesion healed completely at week 20. The median bacterial load (16SrRNA) of BU^+^HIV^+^ patients was 750 copies /ml (95% CI 0–398,000) versus 500 copies/ml (95% CI 0–126,855,500) in BU^+^HIV^−^ group. Similarly, the median count using the IS2404 assay was 500 copies/ml (95% CI 0–500) for BU^+^HIV^+^ patients versus 500 copies/ml (95% CI 500–31,000) for BU^+^HIV^−^ patients. BU^+^HIV^−^ patients mounted a significantly higher interferon-γ response compared to the BU^+^HIV^+^ co-infected patients with respective median (range) responses of [1687(81.11–4399) pg/ml] versus [137.5(4.436–1406) pg/ml, *p* = 0.03]. There were challenges with the integration of HIV and BU care in this cohort.

**Conclusion:**

The prevalence of HIV in the BU+ infected population was not significantly increased when compared to the prevalence of HIV in the general population. There was no clear relationship between BU lesion severity and HIV viral load or CD4 counts. Efforts should be made to encourage the integration of care of patients with BU-HIV coinfection.

## Background

Buruli ulcer (BU), a necrotizing infection of the skin and soft tissues is caused by *Mycobacterium ulcerans* (M. ulcerans)*.* BU has been listed among the Neglected Tropical Diseases (NTD) and is the third most common extra pulmonary mycobacterial disease [1]. Globally, BU is endemic in tropical and semitropical climates with cases reported from 33 countries [2]. In 2015, 2037 new cases were reported worldwide and 94% of these cases were from sub-Saharan Africa [2]. BU can be found in all age groups but in Africa, it is mostly reported in children [2]. The organism produces a toxic macrolide, mycolactone, which has cytotoxic and immunosuppressive properties and is responsible for the extensive destructive lesions which characterize BU disease [3]. Late presentation of the disease can result in large disfigurement, amputations and permanent disability [4].

Human Immunodeficiency Virus infection (HIV) is a global public health issue that has led to the loss of 33 million lives since it was declared an epidemic [5]. Sub-Saharan Africa is the most affected with a reported 25.6 million people living with the disease in 2015. The HIV prevalence among the adult Ghanaian population is 1.7% [6].

In Ghana, the HIV prevalence in the age group 45–49 years is 1.9%; 35–39 years is 3.4% and 15–19 years is 0.7% [7]. In Cameroon, HIV prevalence was 3–6 times higher in BU patients than the regional estimated prevalence (37% vs. 7% in women; 20% vs. 5% in men; and 4% vs. 0.7% in children) [8]. In Benin, BU patients were 8 times more likely to have HIV infection than those without BU (2.6% vs. 0.3%), and in a small cohort in Ghana, HIV prevalence was 5 times higher in BU patients (5% vs. 0.9%) [9].

Reports of the impact of HIV on the clinical presentation and severity of BU disease have indicated an increased incidence of multiple, larger and ulcerated BU lesions in HIV-infected individuals [10]. It also appears that the presence and severity of BU may reflect the level of underlying immune suppression in an HIV-infected person. In Cameroon, 79% of patients with BU category 2 or 3 lesions had a CD4 count of ≤500 cells/mm^3^ compared with 54% of those with category 1 lesions [11].

There is increasing interest in the incidence of BU-HIV coinfection, management strategies applied, and the clinical outcome. Co-infection with HIV is thought to make Buruli ulcer disease more severe, and to negatively affect time to complete healing following treatment [12]. In both diseases, the adaptive immune system is compromised during infection. HIV infects T helper cells (CD4^+^ T cells), dendritic cells and macrophages [13] leading to low levels of circulating CD4^+^ T cells. In BU, mycolactone interferes with T-cell activation, down-regulating expression of the T-cell receptor [14]. Production of various cytokines and chemokines is reduced, and mycolactone treated dendritic cells show reduced ability to activate T-cells [8]. Hence, BU patients produce low levels of interferon-gamma (IFN-γ) [15, 16]. Immune recovery in BU [17] and HIV is driven by the adaptive immune system. Co-infection with HIV complicates the management of patients with BU [18].

The WHO technical team has provided some guidance on how to manage BU-HIV coinfection based on limited data. There is a need for more published data to help guide patient management especially in countries where the disease burden is high [18]. The present study aimed to describe the clinical spectrum of disease and the relationship between HIV viral load and lesion severity in patients with BU-HIV coinfection in central Ghana. We further aimed to assess any differences in the mycobacterial load in BU-HIV coinfected patients compared to age-matched BU patients without HIV infection.

## Methods

### Study design and settings

This was a retrospective cohort study conducted at four BU endemic districts in Ghana from March 2013 to June 2017. The study sites were: Agogo Presbyterian Hospital in the Asante-Akim North District; Nkawie-Toase Government Hospital located in the Atwima Nwabiagya district; Tepa Government Hospital in the Ahafo-Ano district, all in the Ashanti region; and Dunkwa Government hospital in the Upper Denkyira East of the Central region. These sites fall under the network of BU clinics run by a team at the Kumasi Centre for Collaborative Research in Tropical Medicine (KCCR) at the Kwame Nkrumah University of Science and Technology (KNUST) and the Komfo Anokye Teaching Hospital (KATH) in Kumasi, Ghana.

### Study procedures

We retrospectively reviewed the medical records of BU patients who received care in the 4 endemic districts. Patients with confirmed BU who were HIV positive were identified and their BU01 forms were retrieved and reviewed in further detail. Consent for HIV testing was done as part of study procedures during the WHO drug trial for BU [19]. Serum samples were obtained from the patient to screen for HIV after a process of voluntary counselling by trained staff. If the HIV test returned positive, they were subsequently tested for Hepatitis B, and Hepatitis C. HIV positive BU patients were also actively screened for pulmonary tuberculosis.

Plasma samples were obtained for complete blood count and HIV viral load tests at KATH. Viral load and CD4 testing were performed when reagents were available at KATH. Heparinized blood was obtained from BU-HIV coinfected patients (BU^+^HIV^+^) and a matched group of patients with confirmed BU who were HIV negative (BU^+^HIV^−^) for immune response analyses. Cases and controls were matched for age, sex, lesion type and category. In addition, there was a requirement that controls had to have available whole blood stimulated samples for interferon-γ production assessment. The blood samples were taken before and after antibiotic treatment for BU. Swab and fine-needle aspirate (FNA) samples were collected for combined 16S rRNA reverse transcriptase / IS2404 qPCR assay as per standard practice during the drug trial period.

### Treatment

Patients were treated with combination therapy of oral rifampicin (10 mg/kg per day) and intramuscular streptomycin (15 mg/kg per day) or oral rifampicin (10 mg/kg per day) and oral clarithromycin (15 mg/kg per day) for 8 weeks as recommended [20]. Drug administration was under the direct supervision of village health centres to ensure adherence to antibiotic therapy. Patients were scheduled for follow up visits two weekly (during antibiotic treatment) and subsequently monthly at the designated hospitals. Antiretroviral therapy (ART) naïve patients were referred to the Voluntary Counselling and Testing (VCT) centres in the hospitals for further review and provision of ART.

### Wound management within BU clinics

Wounds were assessed using the Aranz Medical Silhouette Camera (Aranz Medical, New Zealand) till the lesions healed. This involved wound measurement, photography and documentation. Wound cleaning was by normal saline; wound dressing involved Vaseline gauze, absorptive dressing material (Beier Drawtex Healthcare Ltd., South Africa) topped with a short-stretched bandage. Frequency of wound dressing varies for patients, depending on lesion size and discharge from the lesions. Pain management during wound care follows the pain ladder protocol of the WHO [21].

### Laboratory methods

#### Buruli ulcer confirmation

Swabs samples obtained from the undermined edges of ulcerative lesions and FNA from non-ulcerative lesions were transported to KCCR in Kumasi for confirmation by PCR targeting the IS*2404*. IS*2404* qPCR was performed by well-established methods as previously described [22–24].

### Combined 16S rRNA reverse transcriptase / IS2404 qPCR assay

The combined 16S rRNA reverse transcriptase / IS2404 qPCR assay was performed as previously described [23, 25]. Briefly, FNA and swab samples were stabilized in 500 μl RNA protect (Qiagen, UK). Whole transcriptome RNA and whole-genome DNA were extracted using the AllPrep DNA/RNA Micro Kit (Qiagen, UK), the RNA extracts were reverse transcribed into cDNA (Quantitect kit) [25]. The cDNA was then subjected to 16S rRNA qPCR and DNA to IS2404 qPCR for quantification of the bacterial load. Ten-fold serial dilutions of known amounts of a plasmid standard of *IS2404* (99 bp) and 16S rRNA (147 bp) (Eurofins MWG Operon, Ebersberg, Germany) were included with PCR amplification for preparation of a standard curve. M. ulcerans bacillary loads in original clinical samples were calculated based on threshold cycle values per template of *IS2404* qPCR (standard curve method) adjusted to the whole amount of DNA extract and the known copy number of 209 *IS2404* copies per M. ulcerans genome on average.

#### Human immunodeficiency syndrome (HIV) diagnosis

Qualitative immunochromatographic (lateral flow) test for HIV (Alere, Japan) was undertaken for each patient. This was to test for the presence of HIV-1/HIV 2 antibodies using plasma samples. Genscreen ELISA test (Bio-Rad Laboratories, France) was later performed for patients who tested positive for HIV1/ HIV2 using plasma samples as routinely done at KATH [26].

#### Hepatitis B and C test

The presence of Hepatitis B surface antigen (HBsAg) and Hepatitis C antibody test (HCV Ab) was tested with plasma samples of HIV positive patients. Hepatitis B lateral flow tests (Inverness Medical, United Kingdom) was performed as shown elsewhere [17]. Hepatitis C lateral flow test (Intec, China) was undertaken as shown elsewhere [27].

#### HIV viral load test

HIV viral load was measured using the AmpliPrep/COBAS TaqMan HIV-1 test system as is routinely done at the serology laboratory of KATH [28] with the output/ result given as the number of copies of viral RNA per millilitre of plasma. Due to programme challenges, viral load and CD4 testing were performed when reagents were available.

#### Whole blood assay

Blood samples were taken at baseline and after treatment for Interferon-gamma (IFN-γ) production assays. At least 6 ml of venous blood was collected in sodium heparin vacutainer tubes (Becton Dickinson, United Kingdom) and transported to the laboratory for diluted whole assay within 2 h of sampling. The whole-blood assay was performed as previously described [29]. Briefly, whole blood was diluted (1 in 10) in RPMI medium supplemented with penicillin [100 IU/ml] and streptomycin [100 g/ml] (Sigma-Aldrich, Germany) in sterile 50 ml Falcon tubes and mixed gently. This was distributed (1 ml per well) into 24-well plates (Nunclon, Denmark) and 5 μg/mL of PMA and 5 μg/mL of M. ulcerans sonicate was pipetted into each well, with one well left unstimulated after which the culture plates were gently swirled ten times clockwise and anticlockwise. Culture plates were incubated at 37 °C in 5% CO_2_ for overnight culture. Supernatants (300 to 500 μl per well) were stored at − 70 °C for IFN-γ enzyme-linked immunosorbent assay.

#### Enzyme-linked Immunosorbent assay (ELISA) and statistical analysis

IFN-γ response was determined with OptEIA sets for ELISA kits for human IFN-γ per the protocol provided by the manufacturer (BD Biosciences, Pharmingen, San Diego, CA) [30]. Optical densities at 450 to 620 nm were measured with an ELISA plate reader (Sunrise Tecan, Austria) with xread plus version 4.30 software. Recombinant cytokine (4.7 to 300 pg/ml; BD Biosciences, Pharmingen, San Diego, CA) was used for the standard curve; the lower detection limit of the ELISA was 4.7 pg/ml. A positive cytokine measurement in the unstimulated culture supernatants, if detected, was subtracted from measurements in the test wells and a standard (best-fit) curve was plotted.

### Statistical analysis

All data were entered in a Microsoft Excel sheet and verified before exporting to the data analysis tool. Data were analyzed with GraphPad Prism 6 software (GraphPad Software Inc., U.S.A). Descriptive and inferential statistical analyses were conducted to present the study data. Categorical variables were expressed as frequencies and proportions, and results for continuous variables were expressed as the median and interquartile range (IQR). HIV prevalence among BU patients was calculated. Descriptive results of cytokines levels of responses of BU with HIV versus BU without HIV, the effect of antibiotic treatment on responses were expressed as medians and ranges. The IS2404 and 16SrRNA results are presented as medians with 95% confidence intervals (95% CI). The medians or means of the data were compared using the Mann-Whitney, or student t-tests and statistical significance was set at *p* < 0.05.

### Ethical statement

Ethical approval for this study was obtained from the Committee on Human Research, Publications and Ethics of the School of Medical Sciences (SMS) at the Kwame Nkrumah University of Science and Technology (KNUST), Kumasi, Ghana [CHRPE/AP/229/12]. All participants provided written informed consent. For participants < 18 years old, consent was obtained from a parent or a legal guardian. The study was conducted in accordance with the ethical principles on research involving human subjects as set out in the Declaration of Helsinki [31]. Sampling and laboratory confirmation for both BU and HIV infection followed the approved national procedures. All confirmed cases were referred for appropriate treatment of BU and HIV.

## Results

### HIV prevalence among Buruli ulcer patients

**Fig. 1:**
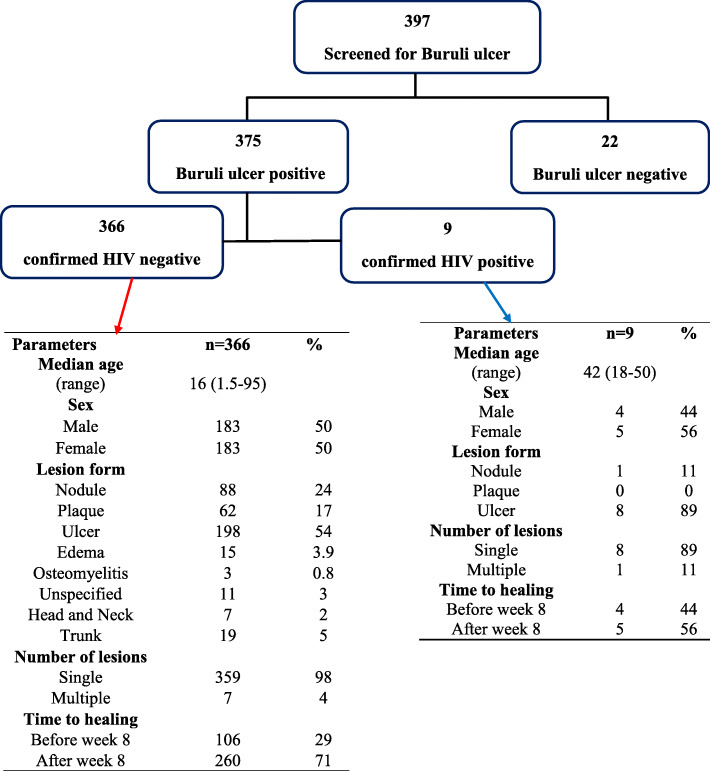
Characteristics of study participants

From March 2013 to June 2017, 397 patients were screened for BU. Ninety-five percent (375 of 397) individuals screened were confirmed to have BU by PCR. Among the PCR-confirmed BU cases, all were tested for HIV and only 9 (2.4%) were found to be co-infected with HIV (Fig. 1). The prevalence of HIV was 2.1 and 2.7% for males and females respectively. No person aged < 18 years had HIV infection in this cohort of BU patients. Two patients (patient 6 and 8) were known with HIV at the time they reported with BU (Table 1). All coinfected patients had HIV-1; there were no patients with HIV-2.

**Table 1:** Demographic and clinical characteristics of BU^+^HIV^+^ co-infected patients

Parameters	Patient 1	Patient 2	Patient 3	Patient 4	Patient 5	Patient 6	Patient 7	Patient 8	Patient 9
Sex	Male	Male	Male	Male	Female	Female	Female	Female	Female
Age (years)	43	42	45	48	50	38	27	40	18
Lesion form	Ulcer	Ulcer	Ulcer	Ulcer	Ulcer	Ulcer	Ulcer	Nodule	Ulcer
Number of lesions	1	2	1	1	1	1	1	1	1
Category of lesion(s)	II	III	II	I	II	II	I	I	II
Lesion site	Upper limb	Lower limb	Lower limb	Lower limb	Back	Lower limb	Lower limb	Lower limb	Upper limb
Lesion healing week	16	52	LF	12	36	20	12	4	4
Occurrence of Paradoxical reaction (PR)	No	No	No PR	No PR	No PR	Yes (at week16)	No PR	No PR	No PR
*Type of PR*	NA	NA	NA	NA	NA	EWP	NA	NA	NA
HIV load (copies/mL)									
*Baseline*	224,856	134	I	255,950	17,538	76,448	ND	ND	ND
*After BU treatment*	1988	LF	LF	1,318,694	140	25,272	60	2,576,972	13,227
CD4 count									
*Baseline*									
*After BU treatment*	130.02				750.3	815.04	753.532	80.6	
Haemoglobin (g/dL)									
*Baseline*	11.7	11	10.3	10.3	9.8	9.8	11.8	8.7	9.7
*After treatment*	12.5	LF	LF	9.3	9.8	9.1	10.7	5.61	ND
Weight (Kg)									
*Baseline*	63	56	50	42	45	66	44	49	52
*After treatment*	62	60	55	44	49	67	46	43	60
Hepatitis B virus	–	–	+	–	–	–	–	–	–
Hepatitis C virus	–	–	–	–	–	–	–	+	–
Buruli ulcer regimen	RS	CR	RS	RS	RS	CR	RS	RS	RS
HIV treatment	3TC, d4T, EFV	not known	not known	not known	3TC, TDF, NVP	3TC, TDF, NVP	AZT, 3TC, NVP	AZT, 3TC, EFV	Not known
Patient on ART before initiation of BU treatment	No	NA	NA	NA	No	Yes	No	Yes	NA

**Abbreviations**: − Negative, + Positive, *RS* Rifampicin and Streptomycin, *CR* Rifampicin and Clarithromycin, *LF* Lost to follow-up, *I* Invalid result, *ND* Not done, *d4T* Stavudine, *3TC* Lamivudine, *NVP* Nevirapine, *TDF* Tenofovir, *AZT* Zidovudine, *EFV* Efavirenz, *PR* Paradoxical reaction, *NA* Not applicable, *EWP* Enlarged, warm to touch lesion

### The spectrum of clinical presentation in BU-HIV (BU^+^HIV^+^) coinfection

Table 1 shows the characteristics of 9 BU^+^HIV^+^ infected patients. Tepa site (5/9) reported the largest number of BU^+^HIV^+^ co-infected patients followed by Agogo (2/9) and Nkawie (2/9). Of the coinfected patients, there were 4 males and 5 females (M/F ratio = 0.8) and their ages ranged from 18 to 50 years (median age = 42). The majority of the lesions were ulcers (8/9) while 1 was a nodule. One patient presented with multiple lesions. Six of the 9 lesions (6/9) were on the lower limb, 2 were located on the upper limb and one lesion was on the back. Category II lesions (5/9) were the most reported followed by category I. Only one patient (patient 6) developed a paradoxical reaction (PR) at week 16. This patient was on ART prior to the diagnosis of BU but the exact duration of ART was not known. The PR was characterized by enlargement of the lesion which was warm to touch and pus-filled. Lesions were followed up until they healed with complete epithelization; 2/9 of the lesions healed before the end of antibiotic treatment.

All patients received antibiotic therapy for 8 weeks as recommended [20]. Seven of nine (7/9) BU^+^HIV^+^ received rifampicin and streptomycin. All BU^+^HIV^+^ coinfected patients received ART but the initiation occurred at different times in different patients due to logistical and programme challenges. Two of the BU^+^HIV^+^ were on ART before the diagnosis of BU but the exact time of initiation of ART could not be ascertained. The other 7/9 of the BU^+^HIV^+^ coinfected patients started ART only after completion of the 8-week antibiotic therapy for BU. Although 4 of the patients confirmed to community-based surveillance volunteers that they had started ART, it was impossible to ascertain the ART regimen in those patients as their ART treatment cards were unavailable for inspection.

There did not appear to be a clear relationship between HIV viral load and the WHO BU categories. All the 3 patients with category II lesions who had viral load testing done had high viral loads (Table 1). The (baseline) viral load was only 134 copies/ ml in the patient with a category III lesion. At baseline, the median (IQR) viral load was 76,448 copies/mL (8836–240,403). After antibiotic therapy viral load levels decreased with a median (IQR) of 13,227 copies/mL (140–1,318,694).

Baseline haemoglobin levels ranged from 8.7 to 11.8 (g/dL) with a median of 10.34 (g/dL). After antibiotic treatment, levels ranged from 5.6 to 12.5 (g/dL) with a median of 9.6 (g/dL). Patients with anaemia received iron and folate supplementation.

### Seroprevalence of hepatitis B and hepatitis C

One patient from the BU^+^HIV^+^ cohort tested positive for HBV and another was HCV positive.

### The interferon-γ response of BU-HIV co-infected patients compared to BU^+^HIV^−^ patients

**Fig. 2:**
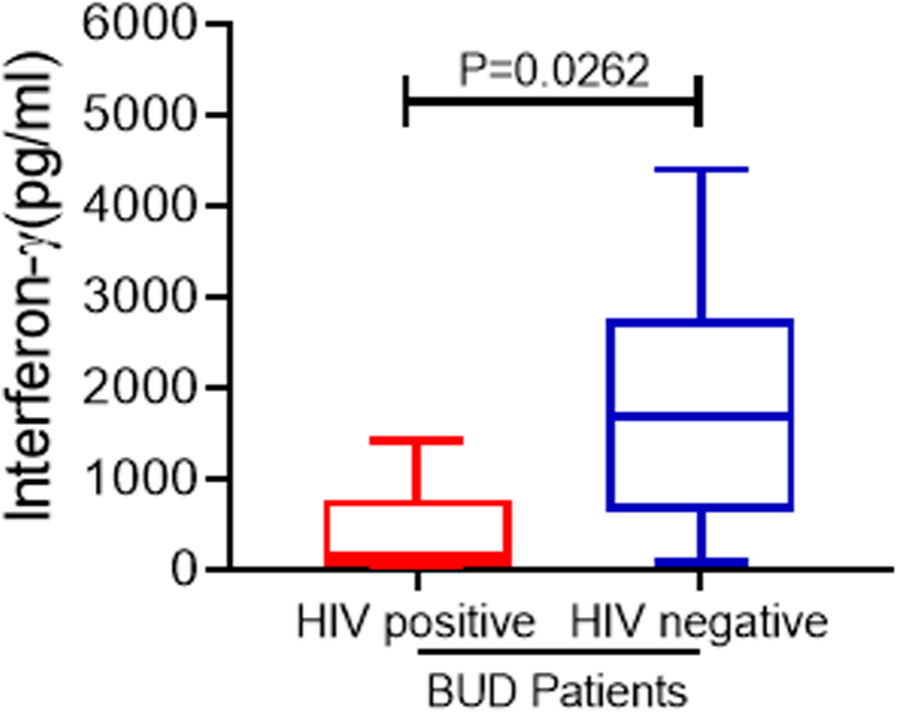
Baseline comparison of interferon-γ responses between BU^+^-HIV^+^ co-infected patients versus BU^+^HIV^−^ patients. Box-and-whisker plots shows interferon-γ (pg/mL) responses of BU^+^HIV^+^ compared to BU^+^HIV^−^ patients at baseline. The box-and-whisker plots were constructed as medians, minimum and maximum values and interquartile ranges. The middle horizontal line represents the median distribution while the lower and upper ends of the box denotes the 25th and 75th percentile of interferon-γ (pg/mL) responses respectively

The results of the interferon-γ responses of BU^+^HIV^+^ and BU^+^HIV^−^ patients at baseline are shown in Fig. 2. The BU^+^HIV^−^ patients mounted a significantly higher interferon-γ response compared to the BU^+^HIV^+^ co-infected patients with respective median (range) responses of [1687(81.11–4399) pg/ml] versus [137.5(4.436–1406) pg/ml, *p*-value = 0.03].

**Fig. 3:**
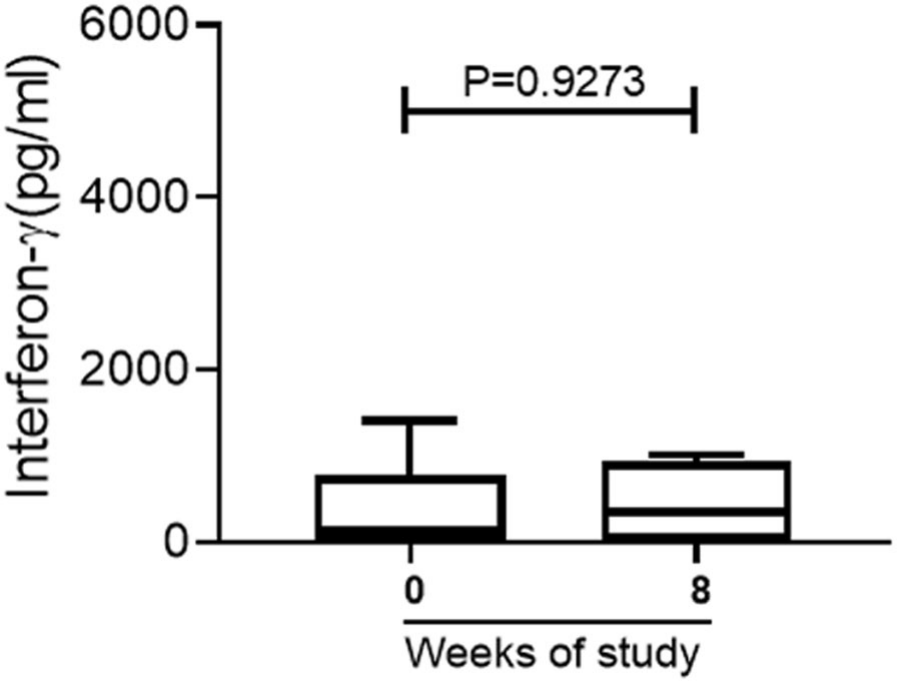
Interferon-gamma responses of BU^+^-HIV^+^ co-infected patients before and after antibiotic treatment. Box-and-whisker plots of interferon-γ (pg/mL) responses of BU^+^HIV^+^ at baseline and after treatment were constructed as medians, minimum and maximum values and interquartile ranges. The middle horizontal line represents the median distribution while the lower and upper ends of the box denotes the 25th and 75th percentile of interferon-γ (pg/mL) responses respectively

There was a trend of increasing interferon-γ response in BU^+^HIV^+^ co-infected patients at week 8 when antibiotic treatment finished [median (range) 346(8.871–1016) pg/ml; 137.511(4.436–1406)] pg/ml pre-treatment] but this was not statistically significant (*p*-value = 0.93) (Fig. 3).

*Mycobacterium ulcerans load in* BU^+^HIV^+^ *coinfected patients compared to BU patients without HIV (*BU^+^HIV^−^).

A median of 750 copies /ml (95% CI 0–398,000) of 16srRNA was detected among the BU^+^HIV^+^ patients compared with a median of 500 copies/ml (95% CI 0–126,855,500) in BU^+^HIV^−^ group. Similarly, the median count using the IS2404 assay was 500 copies/ml (95% CI 0–500) for BU^+^HIV^+^ patients compared to 500 copies/ml (95% CI 500–31,000) for BU^+^HIV^−^ patients (Table 2).

**Table 2:** Comparison of characteristics between BU^+^HIV^+^ patients and BU^+^HIV^−^ patients (age-matched controls)

Characteristic		
	BU^+^HIV^+^ Patients (*n* = 9)	BU^+^HIV^−^ (*n* = 7)
Age, median years (Range)	42 (18–50)	40 (24–51)
Gender, male, n (%)	4 (44.4)	2 (28.6)
Gender, female, n (%)	5 (55.6)	5 (71.4)
Weight, median Kg (IQR)	50 (44, 59)	58 (50, 65)
Duration before seeking treatment, median weeks (IQR)	8 (4,26)	4 (2,10)
**Clinical Forms n (%)**		
Ulcer	8(88.9)	7 (100)
Nodule	1(11.1)	
**WHO Categories, n (%)**		
I (<=5 cm)	3 (33.3)	2 (28.6)
II (5-15 cm)	5 (55.6)	5 (71.4)
III (> 15 cm)	1 (11.1)	
**Location of the lesion, n (%)**		
Lower limb (LL)	6(66.7)	5 (71.4)
Upper limb (UL)	2 (22.2)	1 (14.3)
Other locations	1 (11.1)	1 (14.3)
**Treatment type, n (%)**		
SR8	7 (77.8)	5 (71.4)
CR8	2 (22.2)	2 (28.6)
IS*2404*, median cps/ml (IQR)	500 (500, 500)	500 (500,10,000)
M. ulcerans 16SrRNA, median cps/ml (IQR)	750 (125,83,050)	500 (0, 32,500)
Healing, median weeks (IQR)	14(8,28)	28(12,33)

The median time to healing for BU^+^HIV^+^ coinfected patients was 14 [8–28] weeks while that for the age-matched controls was 28 weeks; however, the difference was not statistically significant (*p* = 0.360).

The time taken for patients to seek medical treatment for the BU lesions was compared among the two groups. Though not statistically significant, a difference in the times was observed as the median time (weeks) it took for the BU^+^HIV^+^ patients to seek treatment was 8 (4–26, IQR) compared to 4 [2–7, 9–11] weeks in the control group (*p* = 0.270).

## Discussion

The median age of 42 years found in this BU-HIV coinfected population is higher than previously reported among coinfected patients from Ghana [13] and Togo [32]. Also, the estimated prevalence (1.7%) of HIV among the general adult Ghanaian population (15–49 years) is higher than in children [33]; this may explain the low prevalence of BU-HIV coinfection in this cohort. The prevalence of HIV in Sub-Saharan Africa is 20–30% [34]. In the current study cohort, the prevalence of HIV was 2.4% which is less than the 4.8% (of 83) reported from a study of BU patients in Togo [32]. The prevalence of BU-HIV coinfection was not significantly differentiated by gender in the current study in contrast to a Togolese study where the prevalence was three times higher in females compared to males [32]. In keeping with the known epidemiology of BU, most BU-HIV patients had their lesions located on the lower limbs.

HIV prevalence in the study population appeared to be comparable to the national HIV prevalence (2.4% vs 1.7% respectively). This is different from the findings in Cameroon [11] where the prevalence of HIV infection in coinfected populations (29%) was significantly higher than the regional estimate. In that study, HIV prevalence among adult patients with PCR confirmed BU was 39 and 17% compared to the regional estimated prevalence of 6.9 and 5.3% for females and males respectively. It is known that some individuals in BU endemic areas develop antibodies to M. ulcerans without any evidence of clinical BU disease [35–37]. Some patients had significant immunosuppression before antibiotic treatment for BU [patients 1, 4 and 8 (Table 1)] since they had high viral load and/or low CD4 counts before and/ or after BU treatment when done. Such patients who have significant immunosuppression when they are exposed to M. ulcerans may be more likely to develop BU disease, but it was difficult to fully investigate this with the limited access to viral load testing and CD4 counts in the current study.

One case of paradoxical reaction (PR) was reported at week 16 (after antibiotic therapy) and healed completely by week 20 in our BU-HIV coinfected patients (patient 6) but in Cameroon, a severe PR was reported 6 months after antibiotic therapy [12]. Both BU and HIV are associated with impaired adaptive immune function which may hinder an early immune response, even though immune reconstitution syndrome which resembles PR, is common in HIV patients beginning antiretroviral therapy. Also, multiple lesions are associated with PR but in this study, only one BU-HIV coinfected patient presented with multiple lesions. High bacterial loads at baseline tend to affect healing and is a risk factor to the development of paradoxical reactions [38]. Our results indicated that though our cohort of BU-HIV coinfected patients had high baseline bacterial loads, it was comparable to that of the age-matched controls without paradoxical reaction episodes. The healing time in both groups was shorter than reported in studies on lesions with high bacterial load [38]. Although the PR developed by patient 6 was also characterised by lesion enlargement and inflammation as was the case in the report by Wanda et al. [12], the PR occurred after completion of antibiotics for BU and the patient was on ART even before specific antibiotic therapy for BU was initiated. It has been suggested that significant baseline immune suppression [12, 39], early commencement of ART [12, 39] and a good response to ART (evidenced by the increase in CD4 counts and suppressed HIV viral load) [12] might be important contributory factors to the development of PR in BU-HIV coinfection. The small sample size of the current study and the challenges to ART initiation made it difficult to ascertain the impact of these factors on the development of PR.

The majority of our study participants presented with single, mostly category II and I lesions. We also identified that 88.9% of the lesions presented by BU^+^HIV^+^ coinfected patients healed after treatment with a median (IQR) healing time of 14 [8–28] weeks. The median time to wound healing in our study is shorter compared to 37 weeks (IQR: 36–37) reported by Tuffour et al [13] and that reported in the recent WHO BU drug trial [19]. This difference in median healing times is most likely because all the 7 patients in the series studied by Tuffour et al. had category III lesions (compared to the current study which had most patients presenting with category II disease). Also, the patients in the study by Tuffour et al. had much lower haemoglobin at baseline compared to those included in the present study and this might have further contributed to the observed differences in the median healing time.

In our study, we could not ascertain a clear relationship between the viral load and the BU lesion severity of the BU^+^HIV^+^ patients at baseline. This was due to number reasons including the very small number of co-infected patients, the difficulty in obtaining baseline viral loads in all BU-HIV infected individuals, the incomplete ART history and incomplete CD4 counts. It was suggested that the severity of BU disease did not necessarily reflect the level of underlying immune suppression, especially when using CD4 as the marker as a case with CD4 counts below 300 had no multifocal disease, while another case with CD4 counts above 500 developed multiple lesions [13]. There are however reports of severe BU lesions like osteomyelitis occurring in patients with severe immune suppression from HIV [13]. Further studies are needed to establish a clearer relationship between BU severity and the level of immune suppression.

In our study, one patient (patient 4) had an increase in the HIV viral load while another (patient 8; no viral load available at baseline) also had a very high viral load after antibiotic therapy. Patient 8 was known with HIV before BU diagnosis but was not adherent to ART. Further questioning revealed the patient doubted the HIV diagnosis, so was re-counselled and tested for reconfirmation followed by subsequent re-enrolment in ART clinic after completion of BU treatment. The very high viral load post BU treatment may be reflective of non-compliance which has the potential to lead to the development of drug-resistant HIV strains. Patient 4 had antiretroviral therapy (ART) initiated rather late due to refusal to enrol in the HIV clinic. The initial challenges encountered with the enrolment of these patients in the ART programme was probably indicative of a significant mental health burden; the assistance of appropriate mental health expert was sought and this helped resolve the difficulties. This also highlights the need to evaluate further the burden of mental health in BU patients especially those with BU^+^HIV^+^ coinfection.

There were challenges with the initiation of ART in the BU^+^HIV^+^ coinfected patients resulting in the start of ART occurring at different times in different patients. Patients had to enrol in HIV clinics which were distant from the BU clinics. Such logistical and programmatic challenges present obstacles for these patients in accessing care and may further hamper adherence to treatment for both BU and HIV infections. Such challenges in the management of BU-HIV coinfection are not peculiar to the current study. In a previous study [13], some patients were referred to ART centres away from the centre of the study only after BU lesions had healed after some 6 months of follow-up because no ART centre was established at the district hospital at the time of management. This calls for efforts to better integrate the care of patients with BU-HIV coinfection.

The prevalence of HIV-HBV and HIV-HCV coinfection in Ghana are 13 and 3.6% respectively [40]. In this study, 1 patient was BU-HIV-HBV positive with another one being BU-HIV-HCV positive. Immune response and haemoglobin levels were not different among the BU-HIV positive regardless of the Hepatitis B or C status before and after treatment in this study. Anaemia was reported among BU-HIV coinfected patients in a previous study [32]. The low haemoglobin levels observed in some BU^+^HIV^+^ coinfected patients after antibiotic treatment for BU was likely related to delayed initiation of ART.

The burden of BU-HIV disease was shown in IFN-γ production where age-matched BU-HIV negative cases mounted significantly higher responses compared to BU-HIV coinfected cases. This difference in IFN-γ responses may be attributed to the impact of HIV on the immune system of BU patients. After BU antibiotic therapy, there was a slightly non-significant improvement in the IFN-γ response of the BU-HIV coinfected patients. It is known in BU immunology that TH1 response improves after treatment [29, 30] therefore HIV infection may have a bigger impact on the overall immune status of the coinfected patients.

The small sample size and inability to measure CD4 counts in patients at baseline notwithstanding, our study contributes important data on BU-HIV coinfection and highlights the need for further research in this area.

## Conclusion

The prevalence of HIV in a cohort of patients with BU in central Ghana was 2.4% but this was not significantly higher than the HIV prevalence in the general population. There was no clear relationship between the WHO lesion category and the HIV viral load. Challenges remain with the initiation of ART in patients with BU-HIV coinfection in Ghana.

## Data Availability

The datasets used and/or analysed during the current study are available from the corresponding author on reasonable request.

## Abbreviations

BU: Buruli ulcer
Mycobacterium ulcerans: M. ulcerans
Human Immunodefiency Virus: HIV
BU^+^HIV^+^: BU-HIV coinfection
BU^+^HIV^−^: BU without HIV infection
IQR: Interquartile range
IFN-γ: Interferon-gamma
KCCR: Kumasi Centre for Collaborative Research in Tropical Medicine
KATH: Komfo Anokye Teaching Hospital
ART: Antiretroviral therapy
VCT: Voluntary Counselling and Testing
FNA: Fine needle aspiration
HBsAg: Hepatitis B surface antigen
HCV Ab: Hepatitis C antibody
PR: Paradoxical reaction

## References

[1] Piersimoni C, Scarparo C (2009). Extrapulmonary infections associated with nontuberculous mycobacteria in immunocompetent persons. Emerg Infect Dis.

[2] WHO (2020). Buruli ulcer (Mycobacterium ulcerans infection): World Health Organization.

[3] Adusumilli S, Mve-Obiang A, Sparer T, Meyers W, Hayman J, Small PLC (2005). Mycobacterium ulcerans toxic macrolide, mycolactone modulates the host immune response and cellular location of M. ulcerans in vitro and in vivo. Cell Microbiol.

[4] Yotsu RR, Suzuki K, Simmonds RE, Bedimo R, Ablordey A, Yeboah-Manu D, Phillips R, Asiedu K (2018). Buruli ulcer: a review of the current knowledge. Curr Trop Med Rep.

[5] WHO (2020). Global Health Observatory (GHO) data-HIV/AIDS: World Health Organization.

[6] COMMISSION GA (2020). Fact sheets and Report (2019).

[7] Ghana NASCP (2016). 2015 HIV Sentinel Survey Report National AIDS/STI Control Programme.

[8] Sarfo FS, Phillips R, Wansbrough-Jones M, Simmonds RE (2016). Recent advances: role of mycolactone in the pathogenesis and monitoring of Mycobacterium ulcerans infection/Buruli ulcer disease. Cell Microbiol.

[9] Christinet V, O'Brien D, Comte E, editors. Management of Buruli ulcer/HIV coinfection: from operational research to WHO guidance: Geneva Health Forum; 2016.

[10] Johnson RC, Ifebe D, Hans-Moevi A, Kestens L, Houessou R, Guédénon A, Meyers WM, Portaels F (2002). Disseminated Mycobacterium ulcerans disease in an HIV-positive patient: a case study. Aids..

[11] Christinet V, Comte E, Ciaffi L, Odermatt P, Serafini M, Antierens A, Rossel L, Nomo AB, Nkemenang P, Tsoungui A, Delhumeau C, Calmy A. Impact of human immunodeficiency virus on the severity of buruli ulcer disease: results of a retrospective study in cameroon. Open Forum Infect Dis. 2014;1(1):ofu021. 10.1093/ofid/ofu021. 10.1093/ofid/ofu021 PMC4324202 25734094

[12] Wanda F, Nkemenang P, Ehounou G, Tchaton M, Comte E, Trellu LT (2014). Clinical features and management of a severe paradoxical reaction associated with combined treatment of Buruli ulcer and HIV co-infection. BMC Infect Dis.

[13] Tuffour J, Owusu-Mireku E, Ruf M-T, Aboagye S, Kpeli G, Akuoku V (2015). Challenges associated with management of Buruli ulcer/human immunodeficiency virus coinfection in a treatment center in Ghana: a case series study. Am J Trop Med Hyg.

[14] McKenna M, Simmonds RE, High S (2016). Mechanistic insights into the inhibition of Sec61-dependent co-and post-translational translocation by mycolactone. J Cell Sci.

[15] Gooding TM, Kemp AS, Robins-Browne RM, Smith M, Johnson PD (2003). Acquired T-helper 1 lymphocyte anergy following infection with Mycobacterium ulcerans. Clin Infect Dis.

[16] Torrado E, Fraga AG, Logarinho E, Martins TG, Carmona JA, Gama JB, Carvalho MA, Proença F, Castro AG, Pedrosa J (2010). IFN-γ–dependent activation of macrophages during experimental infections by Mycobacterium ulcerans is impaired by the toxin mycolactone. J Immunol.

[17] Bieri R, Bolz M, Ruf M-T, Pluschke G (2016). Interferon-γ is a crucial activator of early host immune defense against Mycobacterium ulcerans infection in mice. PLoS Negl Trop Dis.

[18] O'Brien DP, Comte E, Serafini M, Ehounou G, Antierens A, Vuagnat H, Christinet V, Hamani MD, du Cros P (2014). The urgent need for clinical, diagnostic, and operational research for management of Buruli ulcer in Africa. Lancet Infect Dis.

[19] Phillips RO, Robert J, Abass KM, Thompson W, Sarfo FS, Wilson T, Sarpong G, Gateau T, Chauty A, Omollo R, Ochieng Otieno M, Egondi TW, Ampadu EO, Agossadou D, Marion E, Ganlonon L, Wansbrough-Jones M, Grosset J, Macdonald JM, Treadwell T, Saunderson P, Paintsil A, Lehman L, Frimpong M, Sarpong NF, Saizonou R, Tiendrebeogo A, Ohene SA, Stienstra Y, Asiedu KB, van der Werf TS, Osei Mireku S, Abotsi J, Adu Poku JK, Asamoah-Frimpong R, Osei-Wusu B, Sarpong E, Konadu B, Opoku E, Forson M, Ndogyele M, Ofori E, Aboagye F, Berko T, Amofa G, Nsiah A, Mensah-Bonsu J, Ofori Nyarko J, Amoako YA, Koranteng Tannor E, Boakye-Appiah J, Dzibordzi Loglo A, Sarpong-Duah M, Agbavor B, Ardent MF, Yamadjako A, Adanmado Gersande N, Adeye A, Kindjinou M, Akpolan, Kiki M, Sodjinou E, Guegnard C, Klis SA, Velding K, Omansen T, Ofori-Adjei D, Eyangoh S, Knell A, Faber W (2020). Rifampicin and clarithromycin (extended release) versus rifampicin and streptomycin for limited Buruli ulcer lesions: a randomised, open-label, non-inferiority phase 3 trial. Lancet.

[20] Zingue D, Bouam A, Tian RBD, Drancourt M. Buruli ulcer, a prototype for ecosystem-related infection, caused by mycobacterium ulcerans. Clin Microbiol Rev. 2017;31(1):e00045–17. 10.1128/CMR.00045-17. 10.1128/CMR.00045-17 PMC5740976 29237707

[21] WHO. https://www.who.int/cancer/palliative/painladder/en/#:~:text=WHO%20has%20developed%20a%20three,patient%20is%20free%20of%20pain.:

[22] Portaels F, Organization WH (2014). Laboratory diagnosis of buruli ulcer: a manual for health care providers.

[23] Beissner M, Symank D, Phillips RO, Amoako YA, Awua-Boateng N-Y, Sarfo FS, Jansson M, Huber KL, Herbinger KH, Battke F, Löscher T, Adjei O, Bretzel G (2012). Detection of viable Mycobacterium ulcerans in clinical samples by a novel combined 16S rRNA reverse transcriptase/IS2404 real-time qPCR assay. PLoS Negl Trop Dis.

[24] Janssens N, Janicot M, Perera T, Bakker A (2004). Housekeeping genes as internal standards in cancer research. Mol Diagn.

[25] Sarpong-Duah M, Frimpong M, Beissner M, Saar M, Laing K, Sarpong F, Loglo AD, Abass KM, Frempong M, Sarfo FS, Bretzel G, Wansbrough-Jones M, Phillips RO (2017). Clearance of viable Mycobacterium ulcerans from Buruli ulcer lesions during antibiotic treatment as determined by combined 16S rRNA reverse transcriptase/IS 2404 qPCR assay. PLoS Negl Trop Dis.

[26] Iqbal HS, Solomon S, Murugavel K, Solomon SS, Balakrishnan P (2005). Evaluation and diagnostic usefulness of domestic and imported enzyme-linked immunosorbent assays for detection of human immunodeficiency virus type 1 antibody in India. Clin Diagn Lab Immunol.

[27] Hayder I, Ahmed W, Alam SE. Comparison of Different ICT Kits for HBsAg and Anti HCV Using Gold Standard ELISA. Pak J Med Res. 2012;51(3):72–76.

[28] Adjei-Asante K. Developing and applying serological and molecular skills to the virological analysis of HIV-infected patients from Kumasi, Ghana: University of Liverpool; 2013.

[29] Sarfo FS, Phillips RO, Ampadu E, Sarpong F, Adentwe E, Wansbrough-Jones M (2009). Dynamics of the cytokine response to Mycobacterium ulcerans during antibiotic treatment for M. ulcerans disease (Buruli ulcer) in humans. Clin Vaccine Immunol.

[30] Loglo AD, Frimpong M, Duah MS, Sarfo F, Sarpong FN, Agbavor B (2018). IFN-γ and IL-5 whole blood response directed against mycolactone polyketide synthase domains in patients with Mycobacterium ulcerans infection. PeerJ..

[31] World Medical A (2013). World medical association declaration of Helsinki: ethical principles for medical research involving human subjects. JAMA..

[32] Teko M, Salou M, Gbeasor-Komlanvi FA, Konou AA, Ameyapoh Y (2020). Buruli ulcer and HIV Coinfection: cases in Togo. World J AIDS.

[33] WHO. HIV Country Profile 2019-Ghana: World Health Organization; 2019. [WHO/UCN/HSS/19.54:[Available from: https://cfs.hivci.org/country-factsheet.html.

[34] Singh AE, Wong T. Background document: HIV and hepatitis B co-infection: Department of HIV/AIDS, World Health Organization; 2009.

[35] Diaz D, Döbeli H, Yeboah-Manu D, Mensah-Quainoo E, Friedlein A, Soder N (2006). Use of the immunodominant 18-kiloDalton small heat shock protein as a serological marker for exposure to Mycobacterium ulcerans. Clin Vaccine Immunol.

[36] Röltgen K, Bratschi MW, Ross A, Aboagye SY, Ampah KA, Bolz M, Andreoli A, Pritchard J, Minyem JC, Noumen D, Koka E, Um Boock A, Yeboah-Manu D, Pluschke G (2014). Late onset of the serological response against the 18 kDa small heat shock protein of Mycobacterium ulcerans in children. PLoS Negl Trop Dis.

[37] Yeboah-Manu D, Röltgen K, Opare W, Asan-Ampah K, Quenin-Fosu K, Asante-Poku A, Ampadu E, Fyfe J, Koram K, Ahorlu C, Pluschke G (2012). Sero-epidemiology as a tool to screen populations for exposure to Mycobacterium ulcerans. PLoS Negl Trop Dis.

[38] Frimpong M, Agbavor B, Duah MS, Loglo A, Sarpong FN, Boakye-Appiah J, Abass KM, Dongyele M, Amofa G, Tuah W, Frempong M, Amoako YA, Wansbrough-Jones M, Phillips RO (2019). Paradoxical reactions in Buruli ulcer after initiation of antibiotic therapy: relationship to bacterial load. PLoS Negl Trop Dis.

[39] Komenan K, Elidjé EJ, Ildevert GP, Yao KI, Kanga K, Kouamé KA, Abdoulaye S, Hamdam KS, Yao YP, Jean-Marie K (2013). Multifocal Buruli ulcer associated with secondary infection in HIV positive patient. Case Rep Med.

[40] Boateng R, Mutocheluh M, Dompreh A, Obiri-Yeboah D, Odame Anto E, Owusu M, Narkwa PW (2019). Sero-prevalence of hepatitis B and C viral co-infections among HIV-1 infected ART-naïve individuals in Kumasi, Ghana. PLoS One.

